# Performing [^18^F]MFBG Long–Axial-Field-of-View PET/CT Without Sedation or General Anesthesia for Imaging of Children with Neuroblastoma

**DOI:** 10.2967/jnumed.123.267256

**Published:** 2024-08

**Authors:** Lise Borgwardt, Jesper Brok, Kim Francis Andersen, Jacob Madsen, Nicholas Gillings, Marie Øbro Fosbøl, Charlotte Lund Denholt, Ida Nymann Petersen, Louise Sørup Sørensen, Lotte Hahn Enevoldsen, Peter Sandor Oturai, Helle Hjort Johannesen, Liselotte Højgaard, Christina Schulze, Eunice Saxtoft, Flemming Andersen, Barbara Malene Fischer

**Affiliations:** 1 Department of Clinical Physiology and Nuclear Medicine, Copenhagen University Hospital–Rigshospitalet, Copenhagen, Denmark; and; 2 Department of Paediatrics, Copenhagen University Hospital–Rigshospitalet, Copenhagen, Denmark

**Keywords:** pediatrics, [^18^F]MFBG, [^18^F]MFBG LAFOV PET/CT, total-body PET/CT, neuroblastoma

## Abstract

Meta-[^123^I]iodobenzylguanidine ([^123^I]MIBG) scintigraphy with SPECT/CT is the standard of care for diagnosing and monitoring neuroblastoma. Replacing [^123^I]MIBG with the new PET tracer meta-[^18^F]fluorobenzylguanidine ([^18^F]MFBG) and further improving sensitivity and reducing noise in a new long-axial-field-of-view (LAFOV) PET/CT scanner enable increased image quality and a faster acquisition time, allowing examinations to be performed without sedation or general anesthesia (GA). Focusing on feasibility, we present our first experience with [^18^F]MFBG LAFOV PET/CT and compare it with [^123^I]MIBG scintigraphy plus SPECT/CT for imaging in neuroblastoma in children. **Methods:** A pilot of our prospective, single-center study recruited children with neuroblastoma who were referred for [^123^I]MIBG scintigraphy with SPECT/CT. Within 1 wk of [^123^I]MIBG scintigraphy and SPECT/low-dose CT, [^18^F]MFBG LAFOV PET/ultra–low-dose CT was performed 1 h after injection (1.5–3 MBq/kg) without sedation or GA, in contrast to the 24-h postinjection interval needed for scanning with [^123^I]MIBG, the 2- to 2.5-h acquisition time, and the GA often needed in children less than 6 y old. Based on the spirocyclic iodonium-ylide precursor, [^18^F]MFBG was produced in a fully automated good manufacturing practice–compliant procedure. We present the feasibility of the study. **Results:** In the first paired scans of the first 10 children included (5 at diagnosis, 2 during treatment, 2 during surveillance, and 1 at relapse), [^18^F]MFBG PET/CT scan showed a higher number of radiotracer-avid lesions in 80% of the cases and an equal number of lesions in 20% of the cases. The SIOPEN score was higher in 50% of the cases, and the Curie score was higher in 70% of the cases. In particular, intraspinal, retroperitoneal lymph node, and bone marrow involvement was diagnosed with much higher precision. None of the children (median age, 1.6 y; range, 0.1–7.9 y) had sedation or GA during the PET procedure, whereas 80% had GA during [^123^I]MIBG scintigraphy with SPECT/CT. A PET acquisition time of only 2 min without motion artifacts was the data requirement of the 10-min acquisition time for reconstruction to provide a clinically useful image. **Conclusion:** This pilot study demonstrates the feasibility of performing [^18^F]MFBG LAFOV PET/CT for imaging of neuroblastoma. Further, an increased number of radiotracer-avid lesions, an increased SIOPEN score, and an increased Curie score were seen on [^18^F]MFBG LAFOV PET/CT compared with [^123^I]MIBG scintigraphy with SPECT/CT, and GA and sedation was avoided in all patients. Thus, with a 1-d protocol, a significantly shorter scan time, a higher sensitivity, and the avoidance of GA and sedation, [^18^F]MFBG LAFOV PET/CT shows promise for future staging and response assessment and may also have a clinical impact on therapeutic decision-making for children with neuroblastoma.

## Visual Abstract

**Figure:**
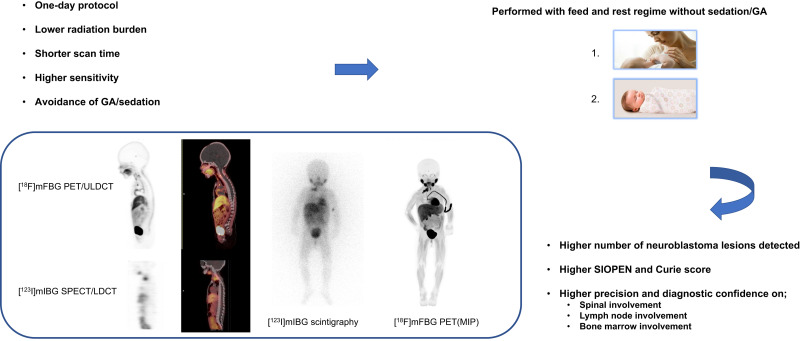

Neuroblastoma is the most common extracranial solid tumor in children, with an overall survival of 70% (1,2). More than 50% of patients present with distant skeletal or bone marrow involvement or soft-tissue metastases at diagnosis, with an overall survival of about 50% (1,2). For decades, scanning with the norepinephrine analog meta-[^123^I]iodobenzylguanidine ([^123^I]MIBG) has been part of the initial staging, response assessment, and follow-up of neuroblastoma. The scanning procedure is a 2-d protocol due to a 24-h tracer uptake period. Sedation or general anesthesia (GA) is often used because of the lengthy scan time (often >2 h), and thyroid-protecting medication is necessary (3). PET is a more sensitive technique than scintigraphy with SPECT/CT; therefore, a shorter scan time and a higher resolution are possible. Some PET radiotracers with different molecular targets have been introduced for neuroblastoma imaging in the past few years, such as [^124^I]MIBG, [^18^F]FDG, [^18^F]F-DOPA, and [^68^Ga]Ga-DOTA peptides, generally identifying a higher number of lesions on PET scans than on [^123^I]MIBG scans (3,4). Meta-[^18^F]fluorobenzylguanidine ([^18^F]MFBG) has a molecular target similar to that of [^123^I]MIBG but is radiolabeled with ^18^F instead of ^123^I and is therefore different from most of the other PET tracers. It is furthermore a 1-d protocol without the need for thyroid-protecting medication (5). Few reports have described the use of [^18^F]MFBG PET/CT in neuroblastomas (5–8). Three clinical studies have yet been published. In the first study of 5 patients with metastasized neuroblastoma, [^18^F]MFBG showed overall physiologic and pathologic distributions similar to those of [^123^I]MIBG, but with detection of additional lesions on [^18^F]MFBG PET/CT (5). The results were confirmed in 20 patients in the second study (6) and in 40 patients in the third study (7), with paired scans in patients with neuroblastoma. In these studies, [^18^F]MFBG PET/CT was performed with an older-generation Discovery 710 PET/CT scanner (GE Healthcare) (5) and on a digital Vision 600 (Siemens Healthineers) (6) and Polestar m660 (Medical Supply Professionals) (7) PET/CT scanner. In our study, we performed [^18^F]MFBG PET/CT on the new long-axial-field-of-view (LAFOV) PET/CT scanner without sedation or GA. The sensitivity of the LAFOV PET/CT scanner is about 10 times higher than that of a digital PET/CT scanner.

We initiated a prospective study to evaluate the diagnostic value and feasibility of neuroblastoma imaging using [^18^F]MFBG LAFOV PET/CT in children compared with [^123^I]MIBG scintigraphy with SPECT/CT. This paper presents the preliminary data and feasibility of the proposed imaging protocol.

## MATERIALS AND METHODS

### Study Design and Participants

This pilot of our prospective, single-center study was performed at Rigshospitalet, Copenhagen University Hospital, and was approved by the local ethics committee (H-21009982; registered at ClinicalTrials.gov, identifier NCT05826158). We recruited the first 10 pediatric patients with suggested or confirmed neuroblastoma who were referred for [^123^I]MIBG scintigraphy with SPECT/CT as part of their regular clinical care (suspicion of disease, staging at diagnosis, or any treatment-response assessment) and their first paired scans. If parents or caretakers gave written informed consent, [^18^F]MFBG LAFOV PET/CT was performed within 1 wk of [^123^I]MIBG scintigraphy with SPECT/CT. Exclusion criteria included being age 18 y or older and pregnancy. The differences in acquisition protocols for [^123^I]MIBG scintigraphy with SPECT/CT in clinical routines compared with [^18^F]MFBG LAFOV PET/CT are listed in Table 1.

**TABLE 1.** Acquisition Protocols for [^123^I]MIBG Scintigraphy with SPECT/CT in Clinical Routine and [^18^F]MFBG LAFOV PET/CT

Parameter	[^123^I]MIBG scintigraphy with SPECT/CT	[^18^F]MFBG LAFOV PET/CT
Acquisition time	∼150 min*	5–10 min
Effective dose of tracer	3.7 mSv	1.7–3.4 mSv
Time from injection to scan	24 h after injection	60 min after injection
Protocol length	2 d	1 d
GA or sedation	Yes^†^	No

* Dependent on length of child (planar + 2 SPECT/CT scans).

† GA or sedation needed for children < 6 y old.

### [^18^F]MFBG Preparation

[^18^F]MFBG was synthesized in our department. The radiosynthesis of [^18^F]MFBG was fully automated using a Synthera+ high-performance liquid chromatography synthesis module (IBA Radio Pharma Solutions) by ^18^F-fluorination and deprotection of the spirocyclic ylide precursor (SPIAd-MBG), in compliance with good manufacturing practices (supplemental materials; available at http://jnm.snmjournals.org).

### [^18^F]MFBG LAFOV PET/Ultra–Low-Dose (ULD) CT

Patients received an intravenous injection of [^18^F]MFBG (1.5–3 MBq/kg) depending on age, with no minimum activity, and without any restrictions in food or medication intake before administration of the radiotracer. Whole-body LAFOV PET/CT was performed on a Biograph Vision Quadra PET/CT scanner (Siemens Healthineers) 1 h after injection (5). To limit radiation use, an ULD CT image was acquired with a fixed tube voltage of 100 kVp and a current modulation of 7 mAs using Siemens CareDose. ULD CT was used for attenuation correction of PET data and large-scale localization. Anatomic localization and correlation with CT were performed with low-dose (LD) CT from the SPECT/CT scan. Whole-body PET was then performed in list mode in 1–2 fields of view (at 106 cm) for 5–10 min per field of view. From previous experience (8), we estimated that a minimum 2-min acquisition time without a motion artifact is required to provide images of clinically acceptable quality with an activity of 3 MBq/kg. The PET procedure was performed without GA or sedation (Table 2). PET images were reconstructed using point-spread-function modeling and time of flight with 4 iterations and 5 subsets, a maximum ring difference of 85, and a gaussian postprocessing filter of 2 mm. The image matrix was 440 × 440, resulting in a 1.65-mm in-plane voxel size. The PET slice thickness was 2 mm, which matched the CT spacing.

**TABLE 2.** Procedures for Avoiding GA and Sedation with [^18^F]MFBG LAFOV PET/CT

Age range	Procedure
0–9 mo	PET/CT planned during nap time
	Parents contacted and procedure and preparation explained at least 1–2 d before scheduled scan
	Sleep and food deprivation, advised starting 1–2 h before scan
	Wrapped, fed, and rested in scanner room
	Scanned without GA or sedation with activity of 1.5 MBq/kg
9 mo to 3 y	PET/CT planned during nap time
	Parents contacted and procedure and preparation explained at least 1–2 d before scheduled scan
	Sleep and food deprivation, advised starting 1–2 h before scan
	Wrapped if possible, light dimmed, fed and rested in scanner room
	Scanned without GA or sedation at activity of 3 MBq/kg*
≥4 y	Parents contacted and preparation explained at least 1–2 d before scan
	Food and drink with lower glycemic index to keep child calm offered in tracer uptake period
	In scanner room, light is dimmed and story is read by parents or caretakers
	Scan performed without GA or sedation at activity of 1.5 MBq/kg

* To be able to reconstruct in only 2 min.

Dosimetry estimation was not performed because it is well described by Pandit-Taskar et al. (5) and Samin et al. (6).

### [^123^I]MIBG Scintigraphy with SPECT/LD CT

Patients were prescribed oral thyroid-protecting medication (potassium iodide for 2 d, starting 1 d before [^123^I]MIBG injection). Scintigraphy images with SPECT/LD CT scans were obtained 24 h after injection of [^123^I]MIBG (European Association of Nuclear Medicine dose calculator: maximum, 200; minimum, 80 MBq) (9) on a Symbia Intevo 16 Bold SPECT/LD CT scanner (Siemens Healthineers). Planar (anterior and posterior) whole-body scintigraphy images were acquired with a 512 × 1,024 matrix size, low- and medium-energy collimators, and a 5 cm/min scan speed. SPECT of the thorax and abdomen and other areas of interest was acquired with a 128 × 128 matrix size, low- and medium-energy collimators, a 15% wide photopeak window centered at 159 keV and similarly sized upper and lower scatter windows, a 20-s acquisition time per view, 64 views per head (90-s views in 2-bed-position tomography), 180 projections in total with step-and-shoot technique for a single bed position and continuous acquisition for 2 bed positions, and a noncircular orbit. An LD CT scan was acquired using 110 kV and 25 mAs, depending on the height and weight of the patient, and 5.0-mm slices. Images were reconstructed using attenuation correction, triple-energy-window scatter correction, and a 4.0-mm gaussian filter, with Flash 3D (Siemens Healthineers), 8 iterations, 4 subsets, and S31 median filter smoothing.

### Image Analysis

Any [^123^I]MIBG or [^18^F]MFBG uptake in bone marrow was regarded as radiotracer-avid lesions of neuroblastoma, and uptake in the soft tissue, not representing physiological uptake, was regarded as radiotracer-avid lesions of neuroblastoma. Planar whole-body scintigraphy, SPECT/LD CT, PET/ULD CT, and maximum-intensity projection images were assessed visually, while taking morphologic data from both LD CT and ULD CT scans into account. Two masked readers independently scored the [^18^F]MFBG scans, and 2 other masked readers scored the [^123^I]MIBG scans for the presence of any pathologic lesions, as the number of lesions in total were counted and the SIOPEN score and the Curie score were calculated. Discrepancies between the pairs of readers were resolved by consensus. All readers were nuclear medicine physicians with more than 10 y of experience in pediatric oncology. The lesions on the paired [^123^I]MIBG and [^18^F]MFBG scans were compared.

### Anti-GD2 Staining

Anti-GD2 staining is not the gold standard, but it is a new and more sensitive method than bone marrow staining, and of all the neuroectodermally derived tumor cell lines and tissues studied by researchers, neuroblastoma is the one known to have the highest expression of GD2 (∼98% expression in all neuroblastoma cell lines, so not just a certain biologic subtype of neuroblastoma cells), estimated at 5–10 million molecules per cell, making it an obvious biomarker for neuroblastoma (10). In our study, GD2 staining was used to confirm bone marrow involvement when the bone marrow staining and [^123^I]MIBG results were negative but the [^18^F]MFBG results were positive.

### Statistical Analysis

Continuous data are presented as median with ranges, whereas categoric data are presented as a percentage. A paired-samples *t* test was applied to assess the differences between the 2 scan methods. Statistical analyses were performed using SPSS, version 24.0 (IBM), with *P* values of less than 0.05 indicating a significant difference.

## RESULTS

[^18^F]MFBG was synthesized (1,600–3,800 MBq) in an overall time of 120 min, with radiochemical yields of 22% ± 8% (*n* = 8). The radiochemical purity was greater than 98%. The product remained stable for at least 6 h at activities of up to 9,500 MBq of [^18^F]MFBG.

Ten children with a median age of 1.6 y (range, 0.1–7.9 y) underwent paired [^123^I]MIBG scintigraphy with SPECT/LD CT and [^18^F]MFBG LAFOV PET/ULD CT. Five children were scanned at the time of diagnosis, 2 during treatment, 2 during off-therapy surveillance, and 1 at verified relapse. The neuroblastoma staging of the children was at diagnosis: *n* = 4 stage L2 (low risk), *n* = 1 stage L2 (intermediate risk), *n* = 1 stage L2 (high risk/relapse), *n* = 2 stage M (high risk), *n* = 1 stage M (high risk/relapse), *n* = 1 stage MS, according to the International Neuroblastoma Risk Group Staging System (Table 3).

**TABLE 3.** International Neuroblastoma Risk Group Staging System

Stage	Definition
L1	Localized tumor without image-defined risk factors for surgery
L2	Locoregional tumor with image-defined risk factors
M	Metastatic
MS	Metastatic in infant < 18 mo old with metastases limited to liver, skin, and bone marrow

**FIGURE 1.**
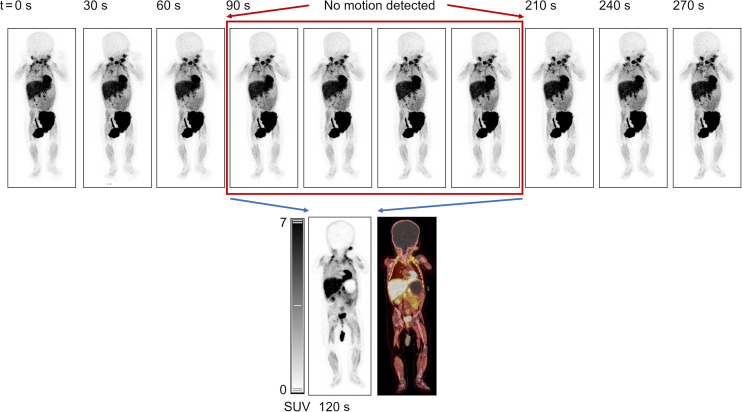
PET images obtained in list mode in 30-s increments, excluding periods of motion artifacts in reconstruction of images.

None of the children underwent sedation or GA during the [^18^F]MFBG LAFOV PET/ULD CT scan, whereas GA was performed in 80% of patients during [^123^I]MIBG scintigraphy with SPECT/LD CT. Only 1 of the 10 children did not fall asleep before or during the [^18^F]MFBG PET/ULD CT scan and was talked to through the procedure by the parents. Two of the 10 children moved during sleep. All PET acquisitions were performed in list mode for 5–10 min. Depending on the age and the expected cooperability of the child, 2-min periods with no movements were identified in these 3 cases and subsequently reconstructed into images of clinically acceptable quality, as illustrated in Figure 1. To be able to identify a 2-min period without motion artifacts, we performed the scans for a longer period than required, as described previously (8), and the utility of this method was confirmed when the lesion counts were compared and found to be equal in all 10 scans with simulated acquisition times of 2, 5, and 10 min. The mean scan time for [^18^F]MFBG LAFOV PET/ULD CT (7.7 ± 2.6 min) was significantly shorter than that for [^123^I]MIBG scintigraphy with SPECT/LD CT (76.3 ± 17.8 min) (*P* < 0.01). The effective dose from CT was, on average, 0.39 mSv (range, 0.36–0.43 mSv) for ULD CT (whole body including the legs) as part of the [^18^F]MFBG PET/CT scan and 1.27 mSv (range, 1.16–2.08 mSv) for LD CT (whole body including the mid thigh) as part of the [^123^I]MIBG SPECT/CT scan. Individual data are available in Supplemental Table 1.

**FIGURE 2.**
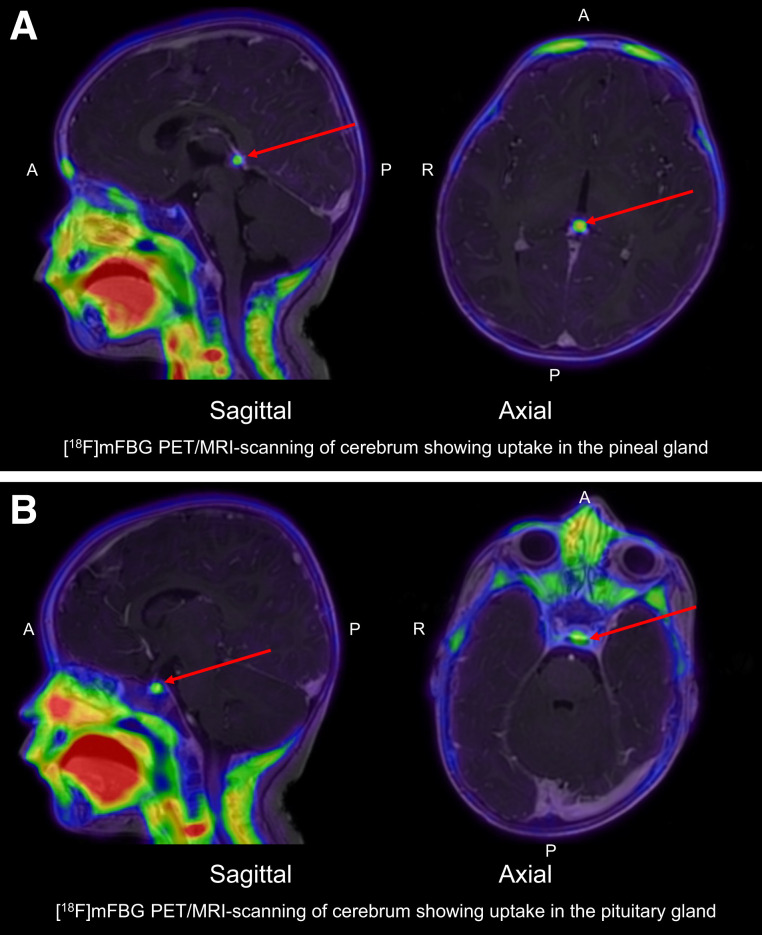
Sagittal and axial view of tracer uptake in pineal gland (arrows, A) and pituitary gland (arrows, B) seen on [^18^F]MFBG LAFOV PET/MRI. A = anterior; P = posterior.

The physiologic uptake observed for [^18^F]MFBG LAFOV PET/ULD CT was similar to the well-known uptake pattern for [^123^I]MIBG scintigraphy (3), such as in the urinary tract, salivary glands, liver, heart wall, adrenal glands, intestines, pancreas, and brown fat; however, uptake in the brown fat, the lacrimal glands, and the pancreas can be more prominent in [^18^F]MFBG LAFOV PET/ULD CT than in [^123^I]MIBG scintigraphy. We also observed physiologic uptake, also seen on follow-up, in the pineal gland (Fig. 2A) in 2 of 10 children when the images were coregistered with MR images (MRI performed for other clinical purposes), as well as discrete physiologic uptake in the pituitary gland (Fig. 2B) in all children. The SUV in structures (SUV_structure_) with physiologic uptake and the SUV ratio (SUV_structure_/SUV_liver_) as the average for [^18^F]MFBG in all 10 patients are shown in Table 4.

**TABLE 4.** SUV of Structures with Physiologic Uptake in [^18^F]MFBG PET Scanning

Structure	SUV	Ratio with liver SUV
Pituitary gland	1.1 (0.4–2.6)	0.3 (0.2–0.9)
Pineal gland	0.7 (0.2–2.1)	0.2 (0.0–0.4)
Lacrimal glands	3.3 (0.8–6.5)	0.9 (0.6–1.3)
Parotid glands	6.4 (3.0–10.6)	2.6 (1.0–4.4)
Submandibular glands	8.4 (4.1–13.1)	2.8 (1.3–5.6)
Thyroid gland	5.0 (1.3–9.9)	1.4 (0.9–2.0)
Heart	6.4 (3.2–9.0)	1.8 (1.1–2.0)
Intestines	2.1 (1.0–2.5)	0.6 (0.5–0.7)
Brown fat	1.6 (1.5–1.7)	0.4 (0.4–0.5)
Pancreas	4.2 (1.0–6.6)	1.2 (0.7–1.8)
Liver	4.0 (3.7–4.9)	

Data are average and range.

**FIGURE 3.**
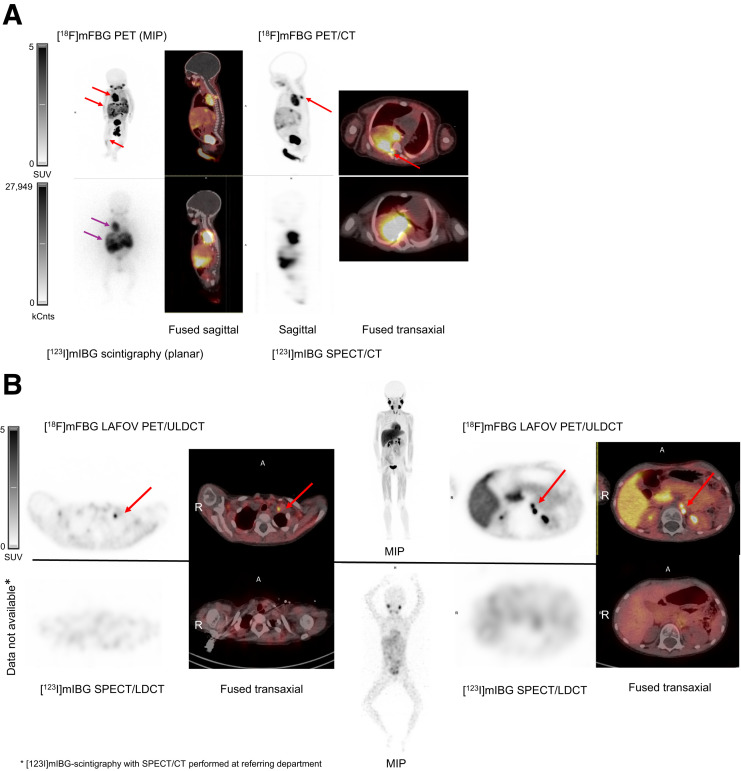
(A) [^18^F]MFBG LAFOV PET/ULD CT (top) and [^123^I]MIBG scintigraphy with SPECT/LD CT images (bottom) of 7-wk-old girl with neuroblastoma. [^18^F]MFBG image shows intraspinal and bone marrow involvement not seen on [^123^I]MIBG image. [^18^F]MFBG PET/ULD CT image shows uptake in tumor in right hemithorax with intraspinal involvement with direct extension into neural foramina and spinal canal between thoracic vertebrae 4/5 and 5/6 (top red arrow), several liver lesions (middle red arrow), and in bone marrow of right tibia (bottom red arrow). [^123^I]MIBG scintigraphy with SPECT/LD CT image shows only large thoracic tumor (top purple arrow) and liver metastases (bottom purple arrow). Spinal involvement and bone marrow involvement could not be identified on [^123^I]MIBG scintigraphy with SPECT/LD CT. (B) [^18^F]MFBG LAFOV PET/ULD CT and [^123^I]MIBG scintigraphy with SPECT/LD CT images of 6-y-old girl with paravertebral relapse of neuroblastoma stage IV in left adrenal gland without N-Myc gene amplification. [^18^F]MFBG image shows lymph node involvement not seen on [^123^I]MIBG image. [^18^F]MFBG PET/ULD CT postoperative image, after resection of paravertebral relapse, shows uptake in left-side cervical lymph node (top left red arrows) and 2 retroperitoneal lymph nodes (top right red arrows), not identified on [^123^I]MIBG scintigraphy with SPECT/LD CT. A = anterior; MIP = maximum-intensity projection.

Compared with [^123^I]MIBG scintigraphy with SPECT/LD CT, [^18^F]MFBG LAFOV PET/ULD CT showed a higher number of radiotracer-avid lesions in 80% of the cases and an equal number in 20% when lesions were counted in the scans. In particular, intraspinal, retroperitoneal lymph node, and bone marrow involvement was diagnosed with much higher precision in [^18^F]MFBG LAFOV PET/ULD CT than in [^123^I]MIBG scintigraphy with SPECT/LD CT (Figs. 3A and 3B). Further, in cases of equivocal [^123^I]MIBG findings, for example, if [^123^I]MIBG scintigraphy with SPECT/LD CT showed suspicion of involvement at these sites, it was diagnosed on [^18^F]MFBG LAFOV PET/ULD CT with a greater diagnostic confidence because of the increased spatial resolution and improved target-to-nontarget contrast of LAFOV PET/CT. When SIOPEN and Curie scores were analyzed and compared, the SIOPEN score was higher in 50% of the cases and the Curie score was higher in 70% of the cases (Table 5).

**TABLE 5.** Comparison of SIOPEN Scores, Curie Scores, and Lesion Counts for [^123^I]MIBG Scintigraphy with SPECT/CT and [^18^F]MFBG LAFOV PET/CT

Patient	Tracer	SIOPEN score	Curie score	Lesion count	[^18^F]MFBG addition
1	[^18^F]MFBG	2	3	3	Bone marrow involvement
	[^123^I]MIBG	0	1	1	
2	[^18^F]MFBG	1	4	4	Intraspinal involvement and bone marrow involvement
	[^123^I]MIBG	0	3	2	
3	[^18^F]MFBG	17	12	21	Bone marrow involvement
	[^123^I]MIBG	0	0	0	
4	[^18^F]MFBG	0	3	2	Confidence in intraspinal involvement
	[^123^I]MIBG	0	3	1	
5	[^18^F]MFBG	0	0	0	No addition
	[^123^I]MIBG	0	0	0	
6	[^18^F]MFBG	4	6	9	Confidence in intraspinal involvement and bone marrow involvement
	[^123^I]MIBG	0	3	2	
7	[^18^F]MFBG	0	2	3	LN cerv. and retro.
	[^123^I]MIBG	0	0	0	
8	[^18^F]MFBG	4	7	8	LN cerv., retro., iliac, and bone marrow involvement
	[^123^I]MIBG	3	6	3	
9	[^18^F]MFBG	0	1	1	No addition
	[^123^I]MIBG	0	1	1	
10	[^18^F]MFBG	0	2	3	LN retro.
	[^123^I]MIBG	0	1	1	

LN = lymph node; cerv. = cervical; retro. = retroperitoneal.

## DISCUSSION

To the best of our knowledge, this is the first demonstration of the feasibility of using [^18^F]MFBG LAFOV PET/ULD CT for imaging of neuroblastoma in children. We have presented the results of paired [^123^I]MIBG and [^18^F]MFBG scans of the first 10 children who were included in our prospective study. [^18^F]MFBG PET/ULD CT scans can be performed without sedation or GA in the most critical age group of 0–4 y because of the possibility of obtaining high-quality PET images, despite short acquisition time, because of the high sensitivity of the LAFOV PET/CT scanner.

We also showed the feasibility of producing tracers in our chemistry laboratory (11–16). These details are provided in the supplemental materials.

None of the children had GA or sedation during the PET/ULD CT scanning procedure. This study demonstrates that close collaboration with the parents before arrival at the clinic and during the procedure, along with prioritizing space with extended time slots on the scanner, can create a calm atmosphere so that GA and sedation can be avoided. Samim et al. (6) reduced use of GA and sedation to 10% (*n* = 20; median age, 4.9 y) because of the shorter scan time, but they were not able to avoid the use of GA or sedation in all patients (the median age in our study was 1.6 y). It might be more difficult to perform PET with diagnostic CT with intravenous contrast, because injection of the intravenous contrast could potentially wake up the sleeping child.

Regarding physiologic uptake, we experienced the same pitfalls for [^18^F]MFBG as for [^123^I]MIBG. This is in accordance with the 3 studies on [^18^F]MFBG PET/CT performed on the older-generation PET/CT (5) and digital PET/CT scanners (6,7). In addition, we found discrete uptake in the pituitary gland and, in some children, also in the pineal gland on [^18^F]MFBG PET/CT, which was not reported earlier. This is most likely due to the increased sensitivity and resolution of LAFOV PET/CT. As both the posterior pituitary gland and the pineal gland are outside the blood–brain barrier (17), this suggests that, as with [^123^I]MIBG (17), [^18^F]MFBG does not seem to cross the blood–brain barrier; however, this needs to be further explored in future studies. We also found that the radiotracer uptake in the pancreas, lacrimal glands, and brown fat may be markedly increased on [^18^F]MFBG PET/CT compared with [^123^I]MIBG scintigraphy with SPECT/CT.

Compared with [^123^I]MIBG scintigraphy with SPECT/LD CT, [^18^F]MFBG LAFOV PET/ULD CT detected more lesions in 80% of the cases and detected an equal number of lesions in the remaining 20% of the patients. This finding suggests that replacing [^123^I]MIBG SPECT/LD CT with [^18^F]MFBG PET/ULD CT will result in higher scores in a significant number of neuroblastoma patients if using either the North American Curie score or the European SIOPEN score (3). In our study, the SIOPEN score was higher in 50% of patients and the Curie score was higher in 70% of patients. Thus, it is important to explore this observation further, as well as to explore the clinical impact in larger studies and consider the potential impact on initial diagnosis, treatment decision-making, treatment-response assessment, and off-therapy surveillance.

It is difficult to define a gold standard, but since [^123^I]MIBG and [^18^F]MFBG are the same molecule, differing only in radiolabel (^123^I and ^18^F, respectively), it is assumed that the increased number of lesions detected when applying the latter radiotracer are true positives found as a result of the increased sensitivity and resolution on PET imaging. We performed the more sensitive GD2 antigen tests in some of the patients with bone marrow lesions seen only on [^18^F]MFBG PET/ULD CT and with negative results on bone marrow aspiration. In all cases, GD2 antigen testing confirmed the uptake in the bone marrow seen on the [^18^F]MFBG scan.

## CONCLUSION

The clinical benefit of [^18^F]MFBG LAFOV PET/ULD CT compared with [^123^I]MIBG scintigraphy with SPECT/LD CT is that it is a 1-d protocol, has a much shorter scan time, and avoids the need for GA and sedation in most patients with neuroblastoma. Preliminary results demonstrate that in most patients, more neuroblastoma lesions were detected on [^18^F]MFBG LAFOV PET/ULD CT than on [^123^I]MIBG scintigraphy with SPECT/LD CT, with increased SIOPEN and Curie scores in 50% and 70% of cases, respectively. [^18^F]MFBG LAFOV PET/ULD CT shows promise for future staging and response assessment in children with neuroblastoma. The impact on staging, treatment decision-making, treatment-response assessment, and off-therapy surveillance remains to be explored.

## DISCLOSURE

No potential conflict of interest relevant to this article was reported.

## Notes

KEY POINTS **QUESTION:** Is [^18^F]MFBG LAFOV PET/CT feasible in a clinical setting, and do we see more lesions on [^18^F]MFBG LAFOV PET/CT than on [^123^I]MIBG scintigraphy with SPECT/LD CT? **PERTINENT FINDINGS:** This study included 10 patients in our prospective study to compare [^18^F]MFBG LAFOV PET/CT with [^123^I]MIBG scintigraphy with SPECT/LD CT in children with neuroblastoma. In total, 80% of [^18^F]MFBG LAFOV PET/CT studies showed more lesions than did studies acquired using [^123^I]MIBG scintigraphy with SPECT/LD CT, and 20% showed the same number of lesions and with increased SIOPEN and Curie scores in 50% and 70%, respectively. [^18^F]MFBG LAFOV PET/CT could be performed without sedation or GA in all patients. **IMPLICATIONS FOR PATIENT CARE:** The clinical benefit of [^18^F]MFBG LAFOV PET/ULD CT compared with [^123^I]MIBG scintigraphy with SPECT/LD CT is that it is a 1-d protocol, has a much shorter scan time, and avoids the need for GA and sedation in most patients with neuroblastoma. The influence of this result for clinical decision-making seems promising.
